# Are We Always Right? Evaluation of the Performance and Knowledge of the Passive Leg Raise Test in Detecting Volume Responsiveness in Critical Care Patients: A National German Survey

**DOI:** 10.3390/jcm13092518

**Published:** 2024-04-25

**Authors:** Eric Hotz, Tisa van Gemmern, Marc Kriege

**Affiliations:** Department of Anaesthesiology, University Medical Centre, Johannes Gutenberg-University Mainz, 55131 Mainz, Germany; eric.hotz@unimedizin-mainz.de (E.H.); tvangemm@students.uni-mainz.de (T.v.G.)

**Keywords:** fluid responsiveness, passive leg raising, stroke volume, critically ill patients

## Abstract

**Background:** In hemodynamically unstable patients, the passive leg raise (PLR) test is recommended for use as a self-fluid challenge for predicting preload responsiveness. However, to interpret the hemodynamic effects and reliability of the PLR, the method of performing it is of the utmost importance. Our aim was to determine the current practice of the correct application and interpretation of the PLR in intensive care patients. **Methods:** After ethical approval, we designed a cross-sectional online survey with a short user-friendly online questionnaire. Using a random sample of 1903 hospitals in Germany, 182 hospitals with different levels of care were invited via an email containing a link to the questionnaire. The online survey was conducted between December 2021 and January 2022. All critical care physicians from different medical disciplines were surveyed. We evaluated the correct points of concern for the PLR, including indication, contraindication, choice of initial position, how to interpret and apply the changes in cardiac output, and the limitations of the PLR. **Results:** A total of 292 respondents participated in the online survey, and 283/292 (97%) of the respondents completed the full survey. In addition, 132/283 (47%) were consultants and 119/283 (42%) worked at a university medical center. The question about the performance of the PLR was answered correctly by 72/283 (25%) of the participants. The limitations of the PLR, such as intra-abdominal hypertension, were correctly selected by 150/283 (53%) of the participants. The correct effect size (increase in stroke volume ≥ 10%) was correctly identified by 217/283 (77%) of the participants. **Conclusions:** Our results suggest a considerable disparity between the contemporary practice of the correct application and interpretation of the PLR and the practice recommendations from recently published data at German ICUs.

## 1. Introduction

Fluid administration is used as a first-line therapy to maintain organ perfusion in patients with acute circulatory failure [1,2,3,4]. Although early fluid administration is beneficial compared with delayed fluid administration, the optimal amount of fluid required for an individual patient varies. The term “optimal” refers to the amount of fluid that restores blood flow to the end organs and, at the same time, does not impair the perfusion of the end organs. In patients with signs of acute circulatory failure, including systemic arterial hypotension, tissue hypoperfusion associated with organ dysfunction, and hyperlactatemia, fluid administration has been historically guided by static indices, such as intravascular pressures (e.g., central venous pressure and pulmonary artery occlusion pressure) and the cardiac output or stroke volume (measured via echocardiography or transpulmonary thermodilution) [2,4]. Concerning its moderate predictive power, the value of the central venous pressure is more useful in gauging the potential risk of further fluid administration than as an accurate predictor of fluid responsiveness in critical care patients [5,6]. 

In contrast, dynamic measures, such as the respiratory dependent diameter of the inferior vena cava (IVC), stroke volume variation (SVV), and passive leg raise (PLR), have been shown to more accurately predict fluid responsiveness in critically ill patients than static parameters, such as central venous and mean arterial pressure [7,8,9,10,11]. The use of the IVC diameter during respiratory changes is limited for obese patients, those undergoing laparoscopic surgery, and those who show a poor echocardiographic subcostal window [12,13]. However, the use of the IVC for decisions about fluid responsiveness should be considered if certain technical and clinical conditions are met, i.e., in patients who are under mechanically controlled ventilation with a tidal volume of ≥8 mL/kg, an intra-abdominal pressure of <12 mmHg in non-obese patients, and in patients without acute cor pulmonale or severe right ventricular dysfunction. Stroke volume variation results from heart–lung interactions and is a sensitive indicator of preload responsiveness. However, a regular heart rhythm and controlled mechanical ventilation with a tidal volume of more than 8 mL/kg of predicted weight (PBW) are required to accurately predict the volume responsiveness [14]. The PLR enables a reversible-volume challenge that is proportional to body size and does not result in volume overload in non-fluid-responsive patients. Furthermore, the advantages of the PLR include the ability to perform the test at the bedside, and it remains reliable in several conditions in which dynamic measures of preload responsiveness that are based on the respiratory variations of stroke volume are limited, such as spontaneous breathing, arrhythmias, tidal volume of <8 mL/kg (PBW), and low lung compliance [15,16]. Monnet et al. described five rules for performing the PLR and the correct application and interpretation of the hemodynamic effects [17]. First, one starts with the correct position of the patient—semi-recumbent (the trunk is at 45 degrees)—followed by lowering the trunk and raising the leg. Second, the hemodynamic effects of the PLR should be assessed solely through the direct measurement of the cardiac output (CO) and not by measuring decreased heart frequency or increased mean arterial pressure [15,16,17]. Third, it is recommended to use techniques such as echocardiography or arterial pulse contour analysis with an effect size of ≥10% of aortic blood flow in real time. Fourth, the measurement of cardiac output should be performed after the PLR when the patient has been removed to the semi-recumbent position. Fifth, confounding factors such as pain when performing the PLR or coughing should be avoided, as these can provoke adrenergic stimulation, resulting in a mistaken interpretation of cardiac output measurements [15,16,17]. 

Adherence to existing recommendations for performing the PLR correctly and the actual implementation of the PLR in German intensive care units (ICUs) have not been studied thus far. We hypothesized that there is high uncertainty regarding the application of the PLR and the interpretation of its hemodynamic effects among critical care patients. Our cross-sectional survey aimed to assess the current practice of the PLR and to identify possible differences in the experiences of the respondents. 

## 2. Methods 

### 2.1. Ethical Considerations

The local ethics committee (Ethical Committee N° 2019-14744) of the medical association of Rhineland–Palatinate State (Chair Dr. A. Wagner) approved the study in 2021. This study was registered with the ClinicalTrial.gov under register number NCT05882240. Data were fully anonymized before the researchers accessed them. A consent form was provided at the beginning of the survey and was circulated among all of the participants via email. The requirement of a written informed consent form was waived by the ethics committee. The study followed the guidelines of the Declaration of Helsinki. We excluded incomplete data reports. Survey links that had been opened without the provision of replies were also excluded from the study.

### 2.2. Study Design and Questionnaire

This study was a qualitative and quantitative analysis of an anonymous cross-sectional mixed-method survey (Lime Survey^®^, Vers. 5.3.19, Hamburg, Germany) in accordance with the STROBE (Strengthening the Reporting of Observational Studies in Epidemiology) [18] guidelines, and it was conducted online from December 2021 to January 2022. 

This was a closed-access survey consisting of 7 introductory questions and 4 questions on the preparation, interpretation, and limitations of the PLR, covering 3 pages. The questions were designed as multiple-choice and multiple-selection questions. The items’ order of appearance was not randomized. Completeness checks were carried out before submission, and the selection of at least one response option was enforced. The survey was targeted to be completed within 10 min. Participants were informed before about the approximate time duration, data storage, data management, and the purpose of the study. The demographic variables included the level of hospital care (first- or second-level hospital or university hospital), the number of ICU beds, the case mix of the unit department (predominantly surgical, medical, mixed, or specialist ICUs, such as for cardiac surgery), as well as the level of training, years of experience, and any additional specialties of the survey participants. Furthermore, in a subgroup analysis, we evaluated the differences between non-cardiac and cardiac surgery ICUs regarding the measurement of the correct PLR effect size.

To identify and explore the current practice in performing the PLR in ICU patients, the survey participants were asked for the correct sequence of patient positions for increasing the test’s sensitivity, followed by questions about the procedure for conducting measurements of the hemodynamic effects and the correct effect size for detecting fluid responsiveness. Finally, participants were also asked about the limitations of the PLR test. To assess the adherence to and knowledge of the correct PLR practice, we created a score for each important point of PLR measurement. A minimum score of 0 (no knowledge of the PLR practice and incorrect statement of the hemodynamic effect size), and a maximum score of 6 (correct PLR measurement) could be achieved (see Appendix A). The questions on the implementation, target value, and effect size of the PLR were scored with one point per correct answer. The question on the limitations of the PLR was awarded three points. The register of the German Interdisciplinary Association for Intensive Care and Emergency Medicine comprised 1340 sites of the 1903 hospitals in Germany reporting their capacity for intensive care beds on a daily basis. The invitation for our online survey was sent to leading ICU physicians of 182 hospitals with different levels of care and reached a response rate of >10%. An email was distributed to each contact who was identified through the aforementioned process (one per institution). The email introduced the nature and the purpose of the survey and invited the contact to complete the survey on behalf of their department. The survey was not advertised publicly. After two weeks, we sent a reminder via email to reach a response rate of >10%. 

### 2.3. Data Analysis 

All collected data were analyzed using GraphPad Prism 9.0 h (GraphPad^®^ Software, Version 9.0 for MAC, La Jolla, CA, USA). 

Descriptive statistics were calculated by providing absolute numbers and percentages of background and demographic variables for all questions relating to the knowledge and current practice of the PLR. The Shapiro–Wilk test was used to examine the distribution of each variable. Normally distributed variables are presented as the mean and standard deviation (SD), while non-normally distributed variables are presented as the median and interquartile range (IQR). Where applicable, contingency tables were produced and analyzed using Fisher’s exact test. Statistical significance was set with a *p*-value of <0.05. 

## 3. Results

### 3.1. Demographics of the Survey Participants

Questionnaires were sent to 182 hospitals with different levels of care. We received 292 responses, and 283/292 (97%) of the complete survey responses were obtained. We excluded nine surveys with incomplete answers on the preparation and interpretation of the PLR in critically ill patients from the analysis (5/9 (55%) of the survey links had been opened without the provision of replies, and 4/9 (45%) of the participants closed the survey after the first question). A total of 46/283 (16%) of the respondents worked at a first-level hospital, 118/283 (42%) worked at a second-level hospital, and 119/283 (42%) worked in a tertiary hospital (university hospital). Of these, 44/283 (16%) worked in mixed ICUs that admitted medical and surgical patients (Table 1). The following distribution of intensive care unit characteristics and the level of training of the survey respondents is shown in Table 1.

### 3.2. Preparation of the Patient Position

A total of 72/283 (25%) of the respondents would start a PLR test intervention with the patient in a semi-recumbent position (with the trunk at 45 degrees), lower the trunk, and raise the leg. However, a proportion of 198/283 (70%) would start with an initial leg raise (both legs) at 45 degrees, and 5/283 (2%) would start with an initial leg raise of one leg. Additionally, 7/283 (3%) would start with a flat position following the leg raise.

### 3.3. Indices of Fluid Responsiveness

A total of 23/283 (8%) of the respondents stated that they would look for a change in the left ventricular velocity time integral (VTI) at the left ventricular outflow tract as measured via echocardiography when conducting the PLR. Other indices are shown in Table 2. Otherwise, most of the participants looked primarily for a change in the systolic pressure (*p* < 0.0001).

### 3.4. Effect Size

A total of 217/283 (77%) of the survey respondents knew the correct effect size for the increased stroke volume (≥10%). Table 3a shows other reported effect sizes. Anesthesiologists responded with the correct effect size (124/283 (80%)) most often, followed by cardiac surgeons 38/50 (76%) and internal medicine physicians 26/42 (62%). Table 3b shows the distribution of the correct effect size with respect to the number of ICU beds. 

### 3.5. Performance and Interpretation of the PLR

A total of 40/283 (14%) of the respondents scored at least four points on the described scale for performing and interpreting the PLR, and no participants achieved the maximum score of six points (correct performance of the PLR, correct effect size, and knowledge about the limitations; also see Appendix A). Table 4 shows the distribution of the points for the correct management of the PLR.

### 3.6. Limitations of the PLR

In the survey, 175/283 (62%) of the participants selected “arrhythmias” as a clinical situation in which it is not appropriate to use the PLR. Table 5 shows the participants’ assessments of other clinical situations in terms of the limitations of the PLR.

### 3.7. Differences between Cardiac and Non-Cardiac Surgery ICUs

A total of 84/283 (30%) of the physicians stated that they worked in a cardiac surgery ICU, compared with 199/283 (70%) in non-cardiac surgery ICU (*p* < 0.0001). In addition, 8/84 (9.5%) of the participants performed VTI measurements with echocardiography, and 36/84 (43%) claimed to evaluate the increased stroke volume with a pulmonary arterial catheter (*p* < 0.0001). In non-cardiac surgery ICUs, 58/199 (29%) of the respondents used the transpulmonary thermodilution method with PiCCO^®^ (Pulse Contour Cardiac Output), compared with 11/84 (13%) in cardiac surgery ICUs (*p* < 0.01). Only 15/199 (7.5%) of the participants stated that they used echocardiography for VTI measurements, and 5/199 (2.5%) considered effect size measurements with pulmonary artery catheters (*p* = 0.03). The proportion of consultants in cardiac surgery ICUs was 34/84 (41%), compared with 98/199 (49%) in non-cardiac surgery ICUs (*p* = 0.19). Consultants more frequently used echocardiography to measure the effect size (23/132 (17.4%)), compared with 0/148 of the trainees (*p* < 0.0001).

## 4. Discussion

The PLR test is a physiological assessment that requires correct and standardized execution. In our survey, only 40/283 (14%) of the respondents scored at least four points on the described scale for performing and interpreting the PLR, with no participants achieving the maximum score of six points (correct performance of the PLR, correct effect size, and knowledge about the limitations). Only 69/283 (24%) used continuous and sufficiently precise cardiac output monitoring, yet 217/283 (77%) were aware of the correct effect size (an increase in VTI/stroke volume by more than 10%). Possible reasons for the inconstancy in the physicians’ responses could be the different case mixes of the ICUs (e.g., mixed ICU versus special cardiac surgery ICU with a focus on hemodynamics), as well as the different levels of experience with a possible lack of teaching following an incorrect understanding of the PLR. Interestingly, small ICUs with 1–10 beds responded with the correct effect size of the PLR more often than ICUs with a higher capacity of beds. There might be an association between the workload of the physicians and the quality of the diagnostic performance of the PLR.

To successfully predict fluid responsiveness, one can use the change in the preload, on the one hand, as well as the measurement of the subsequent changes in physiological variables, such as cardiac output or a derivate-like pulse pressure, on the other hand. The outcome variables of several studies differed from the “flow” variables, meaning the cardiac output or the cardiac index as an assessment of the cardiac output value based on the patient’s size, stroke volume (index), or aortic blood flow. “Pressure” variables, such as pulse pressure, which quantifies the changes in arterial pulse pressure, or systolic pressure, define the difference between the maximum and minimum values of systolic blood pressure following a single positive-pressure breath. Most of the published studies included patients with circulatory failure (mostly sepsis) and had different ventilator modes ranging from mechanically controlled ventilation to spontaneous breathing; most of the included patients had sinusoidal rhythm [15,19,20,21,22,23,24,25,26,27,28]. The measurement techniques varied from transthoracic echocardiography [15,19,20,23,29,30,31], calibrated pulse contour analysis [22,24,25,26,32,33,34,35], and esophageal Doppler analysis [17,19,27] to bioreactance [36,37]. A meta-analysis of 23 clinical trials showed a higher diagnostic performance of changes in flow variables in the PLR (sensitivity of 85% [95% CI, 78–90] and specificity of 92% [95% CI, 87–94]) compared with the PLR-induced changes in pressure variables (sensitivity of 58% [95% CI, 44–70] and specificity of 83% [95% CI, 68–92]; *p* < 0.001) [38]. On the other hand, 50% of ICU patients with acute circulatory failure do not respond to fluid administration, and excessive fluid administration can increase the risk of complications [39]. Therefore, it is very important to measure flow variables (e.g., VTI/stroke volume or cardiac output) in critical care patients. Studies have shown that preferences or familiarity with any measured technique for fluid challenges outside clinical research do not exist. Interestingly, our survey showed consultants’ high familiarity with echocardiography for the prediction of fluid response, but there was no such familiarity in trainees. This might be used as a point of approach for the better teaching of trainees in ICUs. A total of 23/283 (8%) of the respondents identified an increase in stroke volume via an LVOT VTI of ≥10% as predictive of volume responsiveness. However, there are published limitations: (i) VTI measurements are not continuous, and echocardiographic examinations are always dependent on ICU physicians’ experience. (ii) The smallest change in the VTI between two measurements is considered significant and is not attributable to the variability in examinations; even a stroke volume of 11% might be found when the test is performed by the same examiner. (iii) Although this is close to the threshold value required for the PLR (stroke volume increase of at least ≥10%), it is still less precise than a continuous CO measurement using calibrated pulse contour analysis [40] and requires continuous ultrasound examination by an experienced physician, which is not always feasible in clinical practice. 

A total of 41/283 (15%) of the participants considered CO measurement via pulmonary thermodilution using a pulmonary artery catheter (PAC) to be an appropriate monitoring method for predicting volume responsiveness following the PLR. However, this is also a discontinuous method, and the effects of the PLR can be missed if measurements are taken at the incorrect time (as measured with the bolus thermodilution method). Even the modern PAC, which measures CO semi-continuously, only displays an average of measurements from the preceding 3–5 min and, thus, lacks sufficient temporal resolution to accurately assess the PLR [14]. A small yet-to-be-validated study found that a PLR-induced increase in end-tidal carbon dioxide (EtCO_2_) of ≥5% predicted a fluid-induced increase in the CI of ≥15% with 71% sensitivity (95%CI = 48–89%) and 100% specificity (95%CI = 82–100%). The authors concluded that the changes in EtCO_2_ induced using a PLR test predicted fluid responsiveness with reliability, while the changes in arterial pulse pressure did not [35]. A total of 150/283 (53%) of the participants answered correctly regarding the limitations of the PLR. One study demonstrated that raised intra-abdominal hypertension (20 ± 6 mmHg compared with 4 ± 3 mmHg) was responsible for some false negatives in the PLR test [41]. As intra-abdominal hypertension with impaired lung compliance caused by cephalic displacement of the diaphragm is a frequently encountered situation in ICU patients, this limitation gives physicians the opportunity to remember the essential points regarding the PLR.

Further findings corroborated the implementation of pressure variables, such as the pulse pressure variation (PPV). A study from France demonstrated that although 87/145 (60%) of the physicians were familiar with the conditions for measuring the PPV, none were able to accurately interpret the results [42]. A total of 87 of the 145 (75%) of the participants did not perform the PLR in the previously described 45° semi-recumbent position and, instead, raised one or both legs. This approach risks false-positive outcomes due to the activation of the sympathetic nervous system, potentially leading to a subsequent increase in CO [17]. Several requirements limit the accuracy and precision of the diagnostic performance of the PLR in critically ill patients, such as mechanically controlled ventilation versus spontaneous breathing in patients with the corresponding tidal volumes and regular heart rhythm. Other factors, such as the current central volume status and the application of propofol and norepinephrine, influence the degree of preload dependency and, subsequently, the effect of the PLR [24,43,44]. In the case of intraabdominal hypertension, this can possibly provoke increased resistance to venous return. De Backer et al. described that the capacity of PPV is a reliable predictor of fluid responsiveness in mechanically ventilated patients with a tidal volume of at least 8 mL/kg PBW [45]. However, an important drawback of the PPV is that it is inaccurate when using a low tidal volume strategy, which is considered a common ventilation strategy for lung protection in ICU patients. Low tidal volume causes less intrathoracic pressure variation and makes falsely negative PPV-based predictions of fluid responsiveness. Mallat et al. demonstrated in an observational study a poor predictive performance of PPV, which might have been explained through a low tidal volume (median 7.1 mL/kg^−1^ ideal body weight) [46]. Furthermore, they concluded that PLR-induced changes in PPV accurately predict fluid responsiveness with a small grey zone in intensive care patients caused by mechanical ventilation. A meta-analysis of 23 trials showed a similar diagnostic performance of the PLR when comparing spontaneously breathing patients with patients undergoing mechanically controlled ventilation [38]. In addition, no difference was observed when the PLR was performed starting with the recommended semi-recumbent position compared with starting in the supine position. Interestingly, the majority of the patients included in the meta-analysis had a sinusoidal rhythm, so no comparisons between regular heart rhythm and arrhythmias could be made [38]. 

Our study found that the majority of the respondents (99/283 (35%)) considered an increase in systolic blood pressure as an appropriate measure of effect size. However, systolic blood pressure correlates only weakly with CO because the blood pressure is calculated by multiplying the cardiac output by the systemic vascular resistance. It is a clinical reality that not all ICU patients undergoing a PLR test will have access to continuous CO monitoring or any advanced cardiovascular monitoring. In such cases, the pulse pressure (systolic blood pressure–diastolic blood pressure) should be used for assessment rather than the systolic blood pressure [47], although this correlation was not established in other studies [48]. Additionally, increases in diastolic or mean arterial blood pressure did not show a sufficient relationship with the increase in CO in these studies.

## 5. Limitations

Our trial has several limitations. First, although the selection of hospitals was randomized, we had no control over who and how many staff members from a single hospital participated in the survey. This method could have introduced a selection bias. Second, we could not definitively rule out that some participants from one ICU may have taken the survey more than once or that more physicians from one ICU gave their responses. Third, due to a concentration of ICUs that primarily treated surgical patients, there may have been an overrepresentation of this group. Fourth, this study included a limited sample size and the naturally rigid structure of an online survey. This included a moderate response rate that led to a semi-representative sample size. It should also be considered that ICUs with a very high workload were possibly unable to respond to our survey due to a lack of time and resources.

Finally, the findings cannot be extrapolated to those of a national survey, as the number of respondents was inconclusive.

## 6. Conclusions

The PLR test is considered a standard diagnostic procedure for assessing volume responsiveness according to many guidelines. Our study demonstrated that neither the execution nor the interpretation of this seemingly simple test has sufficient accuracy in German intensive care units. There is potential for quality improvement through the education and practical training of ICU physicians. Furthermore, in this context, we should encourage physicians to engage in scientific and medical reading, create local hemodynamic protocols, and establish standard operating procedures in ICUs. 

## Figures and Tables

**Table 1:** Intensive care unit characteristics and levels of training of survey respondents.

Characteristics	Response	%
**Please state the case mix of your ICU**		
Predominantly surgical	94/283	33
Predominantly medical	35/283	12
Mixed/Interdisciplinary ICU	44/283	16
Specialist ICU—Neurological	2/283	<1
Specialist ICU—Cardiac surgery	84/283	30
Specialist ICU—Neurosurgery	13/283	5
Other specialist	7/283	3
N/A	4/283	<1
**Please state the units of staffed beds**		
1–10 beds	48/283	17
11–15 beds	146/283	52
16–30 beds	54/283	19
31–45 beds	14/283	5
Other Units (e.g., Intermediate Care Unit)	17/283	6
N/A	4/283	<1
**Please state your level of training**		
Trainee	148/283	52
Consultant	132/283	47
N/A	3/283	1
Associate Specialist of ICU	90/132	68
**Please state your medical specialty**		
Anesthesiology	154/283	54
Cardiac surgery	50/283	17
Trauma surgery	8/283	3
Abdominal surgery	14/283	5
Neurosurgery	13/283	5
Neurology	2/283	1
Internal medicine	42/283	16

Values are presented as absolute numbers and relative proportions (%).

**Table 2:** Respondents’ indices for correct measurements.

Characteristics	Response	%
**Please state the correct effect size**		
Velocity time integral	23/283	8
PiCCO (Pulse Contour Cardiac Output)	69/283	24
Pulmonary arterial catheter	41/283	15
**Change in Blood Pressure**		
Systolic arterial blood pressure	99/283	35
Mean arterial blood pressure	46/283	16
Pulse pressure variation	4/283	1
N/A	1/283	<1

Values are presented as absolute numbers and relative proportions (%).

**Table 3:** (**a**) Effect sizes for the PLR reported by survey respondents. (**b**) Distribution of the correct effect size with respect to the number of ICU beds.

**(a)**		
**Characteristics**	**Response**	**%**
**Increase of**		
8%	3/283	1
9%	5/283	2
10%	217/283	77
11%	1/283	<1
12%	36/283	12
13%	2/283	<1
14%	0/283	0
15%	16/283	6
N/A	2/283	<1
**(b)**		
**Characteristics**	**Response**	**%**
1–10 beds	41/48	85
11–15 beds	116/146	79
16–30 beds	38/54	70
31–45 beds	9/14	64
Other states	7/17	41

Values are presented as absolute numbers and relative proportions (%).

**Table 4:** Frequency distribution of the points regarding the responses of the participants.

Points	Response	%
0	10/283	4
1	46/283	16
2	84/283	30
3	94/283	33
4	40/283	14
5	9/283	3
6	0/283	0

Values are presented as absolute numbers and relative proportions (%).

**Table 5:** Limitations of the PLR stated by the survey participants.

Characteristics	Response	%
Limitations		
Increased abdominal pressure	150/283	53
Wearing compression stockings	52/283	19
Ventilated patients	11/283	4
Arrhythmias	175/283	62
Traumatic brain injury	141/283	50
Spontaneous breathing	156/283	55
Insufficient tidal volumes in ventilated patients (<8 mL/kg)	62/283	22
Low lung compliance	32/283	11

Values are presented as absolute numbers and relative proportions (%).

## Data Availability

The raw data supporting the conclusions of this article will be made available by the authors without undue reservation.

## Appendix A. Questionnaire

**Questions:**	**Points**
1. You would like to test the volume responsiveness of your patient using the passive leg raise (PLR) test. How do you proceed?	0
(a) Raise both legs above 45°	0
(b) Raise one leg above 45°	0
(c) First, position the patient in a flat position, and then do so in a head-down position (Trendelenburg)	0
(d) Raise the upper body to 45° with subsequent tilting of the bed so that the trunk is flat and the legs are raised	1
(e) None of the above	0
2. Which target value do you use to assess the effect of the PLR?	
(a) Increase in systolic blood pressure	0
(b) Increase in mean arterial blood pressure (MAP)	0
(c) Increase in pulse pressure (systolic—diastolic blood pressure)	0
(d) Increase in cardiac output by means of continuous cardiac output measurement (e.g., PICCO)	1
(e) Increase in cardiac output using discontinuous cardiac output measurement (e.g., LVOT VTI using echocardiography)	0
(f) Increase in cardiac output by means of discontinuous HZV measurement (e.g., using PAK)	0
(g) None of the above	0
3. What effect size of the target variable that you have assessed do you expect the patient to respond to?	
(a) 8% increase	0
(b) 9% increase	0
(c) 10% increase	1
(d) 11% increase	0
(e) 12% increase	0
(f) 13% increase	0
(g) 14% increase	0
(h) 15% increase	0
(i) None of the above	0
4. What are the clinical situations in which the PLR is not useful? (multiple answers possible)	
(a) Increased intraabdominal pressure	1
(b) Wearing compression stockings	1
(c) Ventilated patients	0
(d) Arrhythmias	0
(e) Traumatic brain injury	1
(f) Spontaneous breathing	0
(g) Insufficient tidal volumes in ventilated patients (<8 mL/kg)	0
(h) Low lung compliance	0
In total	=6 Points
Further Questions:	
5. You are:	
(a) Doctor in residence	
(b) Specialist doctor	
6. Your function is:	
(a) Assistant doctor	
(b) Functional senior physician/deputy senior physician	
(c) Consultant	
(d) Senior consultant	
(e) Chief physician	
7. Your specialty is: (If more than one applies, please select your current specialty)	
(a) Internal medicine	
(b) General surgery	
(c) Orthopedics, trauma surgery	
(d) Pediatrics	
(e) Anesthesiology	
(f) Neurology	
(g) Neurosurgery	
(h) Other specialty	
8. Do you have the additional qualification “Special Intensive Care Medicine”?	
(a) Yes	
(b) No	
9. Your current place of work is: (If more than one applies, select your current specialty):	
(a) Intensive care unit with a bed capacity of 1–10	
(b) Intensive care unit with a bed capacity of 11–15	
(c) Intensive care unit with a bed capacity of 16–30	
(d) Intensive care unit with a bed capacity of 31–45	
(e) Intensive care unit with a bed capacity of 46–60	
(f) Intermediate care unit	
(g) Recovery ward	
(h) Functional area	
(i) Other location	
10. You mainly care for patients in the following specialties:	
(a) Interdisciplinary	
(b) Post-operative patients (mixed collective)	
(c) Cardiac surgery	
(d) Pediatrics	
(e) Internal medicine	
(f) Neurosurgery	
(g) Neurology	
(h) General surgery	
(i) Orthopedics, trauma surgery	
(j) Other specialty	
11. You work in a clinic of the care level: (Please select the most appropriate.)	
(a) Basic and standard care	
(b) Maximum care	
(c) University hospital	
